# Vanishing Pseudotumoral White Matter Lesions Presenting as Aphasia and Altered Mental Status in a 71-Year-Old Male

**DOI:** 10.7759/cureus.6284

**Published:** 2019-12-03

**Authors:** Daniela Ferro, Mafalda Seabra, Isabel Taveira, Carina Reis, Joana Guimarães

**Affiliations:** 1 Neurology, Centro Hospitalar São João, Porto, PRT; 2 Internal Medicine, Hospital Do Litoral Alentejano, Santiago do Cacém, PRT; 3 Neuroradiology, Centro Hospitalar São João, Porto, PRT

**Keywords:** tumefactive demyelinating disease, late onset adem, pseudotumoral lesions

## Abstract

Acute disseminated encephalomyelitis (ADEM) is a demyelinating disorder that usually affects the central nervous system (CNS) after an infection and/or vaccination. It is more common in infancy. Here we present a case of late onset ADEM.

A 71-year-old male was admitted to the emergency department due to speech difficulty and somnolence. Upon neurological examination he had a mixed aphasia. He performed a brain computed tomography which showed multiple white matter hypodense lesions. After admission to the neurology ward, he performed a lumbar puncture which showed a mildly inflammatory cerebrospinal fluid, negative serological testing and negative oligoclonal bands. Brain magnetic resonance imaging (MRI) confirmed the presence of multiple T2 hyperintense lesions, extensive bilateral frontoparietal lesions with abundant perilesional edema, four with gadolinium enhancement in an open-ring pattern and no mass effect. Anti-aquaporin 4 antibody, virologic and bacteriologic blood testing, screening of autoimmune disorders and occult neoplasm were all unremarkable. He was treated with intravenous methylprednisolone (1 gr) during five days and started to recover, maintaining a slight verbal fluency deficiency. Post-treatment brain MRI showed reduction of previous lesions, corroborating the probable inflammatory/demyelinating etiology. After discharge he maintained follow-up at the neurology outpatient clinic and he is currently asymptomatic with no new lesions and further reduction of the previous ones on follow-up MR scan.

Both clinical follow-up of the patient, revealing a monophasic course with complete recovery, and temporal evolution of his brain lesions were essential to establish a diagnosis of ADEM in a septuagenarian patient, in whom other diagnoses have to be considered.

## Introduction

Demyelinating disorders of the central nervous system (CNS) such as multiple sclerosis (MS) and acute disseminated encephalomyelitis (ADEM) are pathologies that usually begin in childhood or early adulthood [1]. ADEM is a rare disorder characterized by a monophasic course and usually does not require additional immune-modulating treatment after resolution of the acute phase. On the other hand, MS is a relapsing disease that benefits from the institution of long-term immunomodulatory therapies in order to halt disease progression. After a single episode of neurological deficits, only follow-up will indicate if there will be progress to a relapsing disease such as MS. In both cases, the disease rarely initiates after the age of 50 [2,3]. Series of adult-onset ADEM patients have shown that this condition can arise in patients as old as 82-year-old, although the most common age of onset in adult patients is the 4th decade of life [4-6]. In most patients, ADEM is related to a good clinical prognosis, but the outcome in the elderly is more difficult to define regarding the paucity of cases described in the literature. A study comparing clinical presentation and outcome of ADEM in children versus adult patients found that outcome of ADEM is more severe in adults with respect to hospitalization, intensive care unit admission, recovery and mortality [3]. We present a case of ADEM in a 71-year-old man, who recovered completely after corticotherapy and discuss some of the clinical and radiological aspects that helped to distinguish ADEM from MS in this particular case.

## Case presentation

We present the case of a retired 71-year-old man complaining of a sudden onset speech difficulty and somnolence.

The patient first came to our emergency department (ED) five days after onset of the symptoms and on neurologic examination presented a mixed aphasia and a right central facial palsy. He had a history of headache after a dental procedure performed one month earlier, which resolved with ibuprofen within five days. No history of fever, malaise, loss of appetite or weight, respiratory or urinary complaints was present at this point. He had a medical history of hypertension, dyslipidemia, previous smoking and glaucoma and his regular medications consisted of common drugs to treat his comorbidities (enalapril + lercanidipine p.o., simvastatin p.o., lorazepam p.o., sucralfate p.o., latanoprost + timolol ophthalmic drops). His younger sister died of leukemia (not-otherwise specified by the patient) at the age of 29. At the ED, the patient performed a brain head computed tomography (CT) scan which revealed multiple hypodense lesions in the white matter, mainly in the frontal and parietal lobes, with no mass effect or enhancement with iodinated contrast of unclear etiology. Therefore, the patient was admitted to our neurology ward for further investigation.

**Figure 1:**
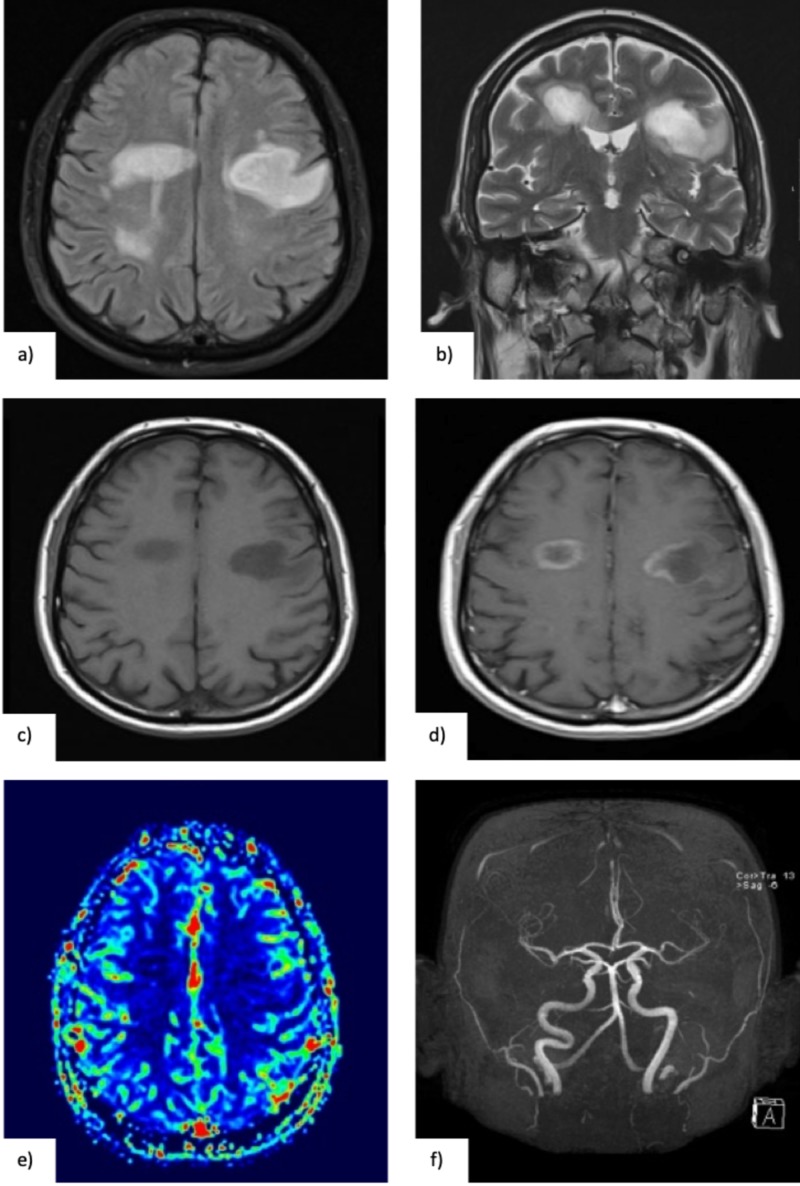
(a, b) Brain MRI (14-06-2017), showing white matter lesions, hyperintense in T2 FLAIR (axial) and T2 TSE (coronal) with surrounding oedema. (c) These lesions were hypointense in T1 (axial), and (d) presented open-ring enhancement after gadolinium injection. (e) Perfusion sequences revealed no increased perfusion in the mentioned lesions and (f) MRI angiogram was normal.

A more detailed general examination revealed multiple skin nevi and he was consulted by a dermatologist who concluded that there were no signs of malignancy. He had no palpable lymph nodes in the cervical, clavicular, axillar or inguinal ganglion chains. He performed a brain magnetic resonance imaging (MRI) scan, perfusion MRI and magnetic resonance angiography (MRA), which revealed multiple bilateral T2 hyperintense and T1 hypointense frontoparietal lesions, surrounded by abundant edema. Four of them had gadolinium enhancement, with no areas of increased perfusion (Figure 1).

The investigation was complemented with cerebrospinal fluid (CSF) study which showed eight white blood cells/mm^3^ and mildly elevated protein concentration (0.60 g/L) with negative serum and CSF oligoclonal bands. Cytology for malignant cells and serologies for *Borrelia burgdorferi*, *Mycobacterium tuberculosis*, *Treponema pallidum*, *Toxoplasma gondii*, *Nocardia* spp. were all negative in CSF sample. Blood tests showed only a slightly elevated sedimentation rate (44 mm in the first hour) and systemic occult neoplasm screening was unremarkable, including serum electrophoresis and immunoglobulin concentrations, cervico-thoraco-abdomino-pelvic CT, abdominal echography, endoscopic examination of the stomach and colon and a gallium whole body scintigraphy. Other blood tests such as angiotensin converting enzyme levels, screening for serological tests related to auto-immune disorders (antibodies against granulocyte cytoplasm, antinuclear, anti-Ds DNA, antiphospholipid and anti-extractable nuclear antigen antibodies and rheumatoid factor’s levels) and blood testing for human immunodeficiency virus type 1, hepatitis B virus, hepatitis C virus, Epstein-Barr virus, *Borrelia burgdorferi*, *Brucella* spp. and *Treponema pallidum* were all negative. The interferon gamma release assay (IGRA) test was performed since there was a possibility that the patient would need some kind of immunosuppression and considering that there is a high prevalence of tuberculosis in Northern regions of Portugal. A positive result was obtained and the patient was then evaluated by an infectiologist who concluded that there had been a previous contact with Mycobacterium tuberculosis with no evidence of current infection. The patient completed a nine-month course of prophylactic treatment with isoniazid. Blood testing for antibody anti-aquaporin 4 and anti-myelin oligodendrocyte glycoprotein, through cell-based assay, was also negative.

**Figure 2:**
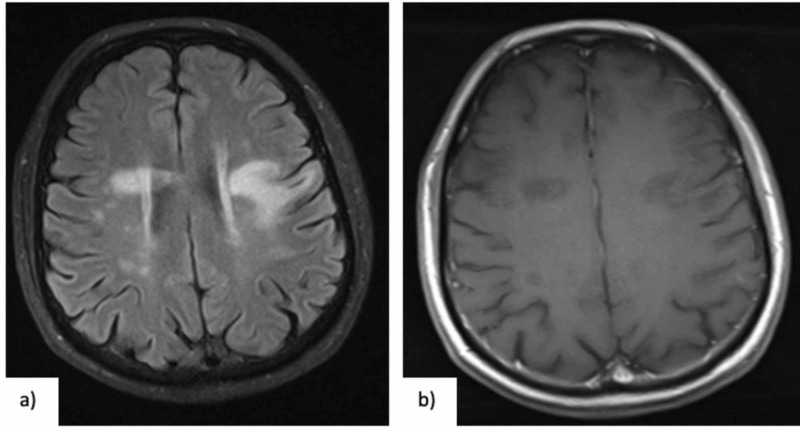
(a) Brain MRI (05-07-2017) performed after corticotherapy, showing reduction of previous lesions, in T2 FLAIR (axial), and (b) correspondent reduction of hypointense lesions in T1 (axial), with almost no gadolinium enhancement.

The patient gradually started to improve without treatment, although he still maintained at this point an altered speech fluency reflecting a remaining language deficit. A second brain MRI (performed 20 days after the first scan) showed a slight decrease in lesion size and edema but still some gadolinium enhancement. A multidisciplinary discussion with infectiologist and neuroradiologist was done and after excluding infectious disorders which could aggravate with corticosteroid treatment and considering the possibility of demyelinating inflammatory etiology for the brain lesions, treatment with five days of intravenous methylprednisolone was attempted, with no clinically manifested adverse effects. A clinical and imagiologic re-evaluation was done 15 days after the course of corticosteroids. At this point, the patient had already improved verbal fluency and no other focal deficits at neurological examination and brain MRI showed an improvement in lesion size, along with reduced edema and nearly no gadolinium enhancement at this point (Figure 2).

Whole spinal cord MRI was also performed and no spinal lesions were found.

**Figure 3:**
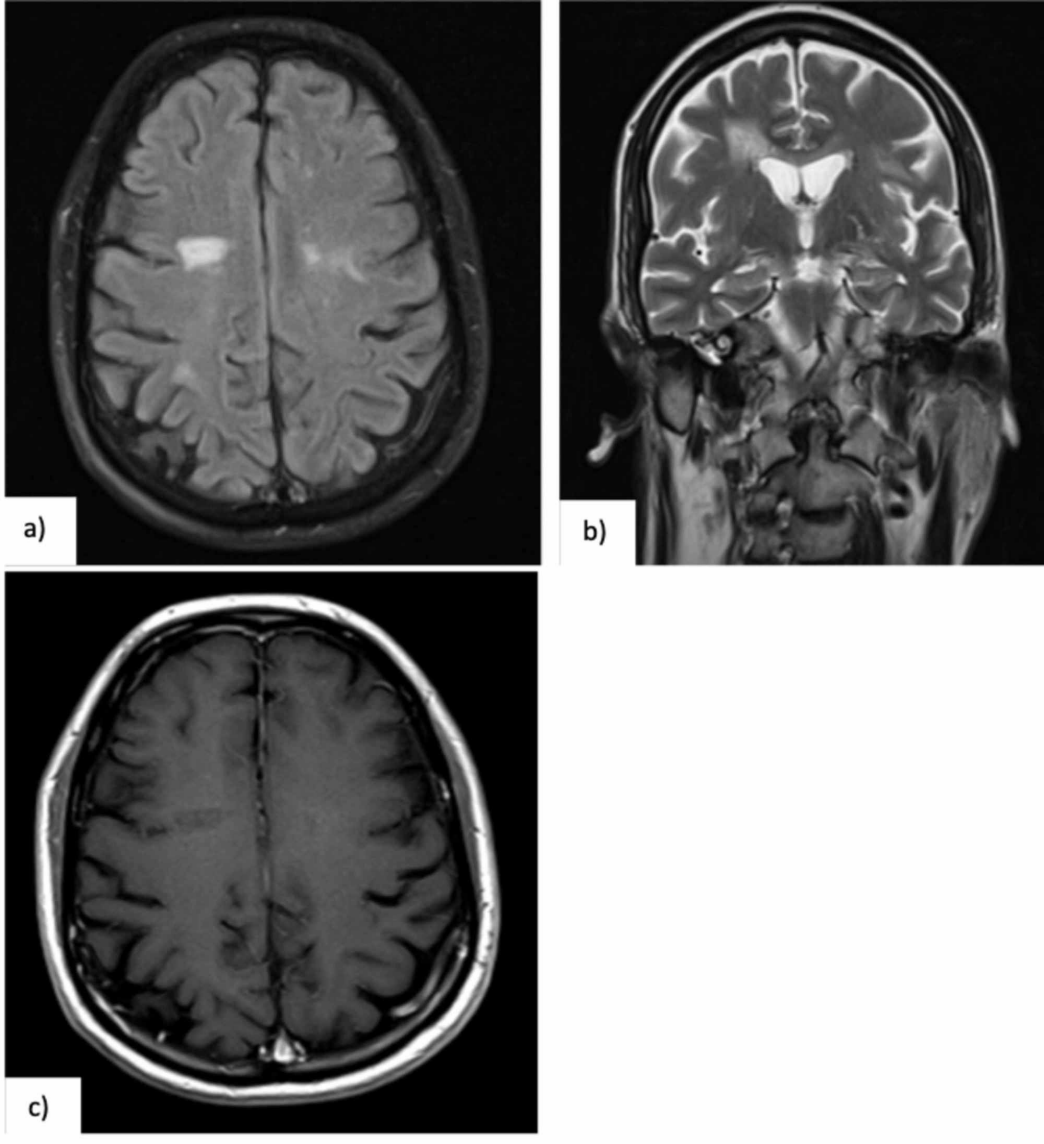
(a, b) Brain MRI (04-06-2018) performed one year after clinical presentation, showing remarkable reduction of previous lesions in T2 FLAIR (axial) and T2 TSE (coronal) and sequences, and (c) almost no perceptible T1 hypointensities (axial, after gadolinium injection).

After 26 months of surveillance with no additional treatment or recurrence of symptoms, the patient has no clinically perceptible deficit and last MRI is almost unremarkable (Figure 3).

## Discussion

White matter lesions can be found at presentation in many CNS or systemic disorders. Due to the patient’s age, we thought first to be important to exclude neoformative process involving the CNS (primary or metastatic). Metastatic lesions were excluded with an extensive systemic screening, both blood and CSF testing and imagiologic exams; CNS primary tumor was unlikely due to the spontaneous recovery of the patient (both clinically and imagiologically) as well as specific imaging findings such as a lack of increased perfusion of the lesions in MRI examination.

A second important diagnosis to be ruled out was an infectious etiology, as the prompt diagnosis and management of infectious spreading would be determinant of the prognosis. An extensive blood and CSF workup allowed us to exclude this possibility and, retrospectively, this infectious etiology proved to be unlikely once the patient started to respond favorably to corticosteroid therapy. The multidisciplinary discussion with other specialties was of crucial importance to exclude these diagnoses and to finally define the most probable demyelinating disorder related with the patient’s lesions.

Since the anti-aquaporin 4 antibody was negative and the imaging studies were atypical for neuromyelitis optica spectrum disorders (including no previous evidence of optic nerve or spinal cord lesions), we focused our attention on the differential diagnosis of late-onset ADEM and late-onset MS. ADEM often presents after an infection or vaccination and in this case we could not find evidence of infection other than a history of headache without fever after a dental procedure performed one month earlier, as mentioned. Although these symptoms seem insufficient to assume an infectious etiology, we must not disregard the fact that the patient started anti-inflammatory drug intake at symptom onset. On the other hand, the initial symptoms (aphasia and altered mental status) were more suggestive of ADEM than MS, which often presents with a monosymptomatic motor or sensory deficit. As for the imaging characteristics, the large, poorly defined tumefactive lesions, without evidence of previous disease activity, favor the diagnosis of ADEM considering that MS lesions are often small, ovoid, periventricular or juxtacortical lesions [7-10]. Additionally, the diagnosis of MS could not be given at this point since no dissemination in space, time or presence of oligoclonal bands were observed [11]. In both situations, open-ring enhancement after gadolinium seems to support a demyelinating aetiology [12]. The characteristics that were considered in differentiating between these two entities are summarized in Table 1.

**Table 1:** Clinical, MRI and CSF characteristics considered in the differential diagnosis between MS and ADEM + the specific feature is more frequently found in the mentioned diagnosis; - the specific feature is less frequently found in the mentioned diagnosis. ADEM: Acute disseminated encephalomyelitis; CSF: Cerebrospinal fluid; MS: Multiple sclerosis.

		Multiple Sclerosis	ADEM	Patient
Clinical features	Age	Young adults	Children	71
	Prodromal phase	-	+	No
	Clinical characteristics	Monosymptomatic	Polysymptomatic (including encephalopathy, aphasia, bilateral optic neuritis, seizures)	Aphasia + encephalopathy
	Clinical course	Relapsing-remitting (>3 months after)	Monophasic	Monophasic
MRI findings	Margins of lesion	Defined, ovoid	Poorly demarcated	Poorly demarcated
	Size of lesions	Small, <1 cm	Large, >1-2 cm	Large, pseudotumoral
	Evidence of previous lesions	+	-	No
	Deep grey matter involvement	-	+	No
	Periventricular lesions	+	-	No
CSF	Oligoclonal bands	+	-	No

Even though these characteristics favored the diagnosis of late-onset monophasic ADEM at an early stage, this diagnosis was only possible after a period of observation after which the patient remained stable and with no further clinical or radiological activity. Late-onset MS seems to be related to a higher probability of primary progressive disease rather than a relapsing-remitting course as well as a rapid progression to secondary progressive phase from onset [13]. Considering the advanced patient’s age at onset and the absence of new neurological symptoms or signs at this point, ageing will likely reduce the relapse risk in this case.

Fortunately, our patient recovered after five days of corticotherapy which allowed us to halt immunomodulatory treatment at this time. A diagnosis and treatment algorithm proposed by Algahtani et al. for tumefactive demyelinating lesions management presents the way we would probably have proceeded if no recovery was observed and no additional data contradicted the assumed diagnosis of ADEM (escalating therapy to IV immunoglobulin, plasmapheresis and rituximab) [14].

## Conclusions

This case illustrates the difficulty that sometimes arises when distinguishing demyelinating disorders affecting the CNS, particularly at an initial approach and in atypical ages of onset. We find it important to first exclude other etiologies such as infectious and neoplastic disorders that would need a different treatment. After excluding these conditions, we initiated treatment with corticotherapy to halt the likely immune-mediated mechanism behind the patient’s lesions, although no clear diagnosis was made at this time, considering that waiting too long could lead to permanent neurological deficits. We also emphasize the importance of a multidisciplinary discussion and a stepwise decision in the approach of these disorders.
